# Integration between Glycolysis and Glutamate-Glutamine Cycle Flux May Explain Preferential Glycolytic Increase during Brain Activation, Requiring Glutamate

**DOI:** 10.3389/fnint.2017.00018

**Published:** 2017-08-25

**Authors:** Leif Hertz, Ye Chen

**Affiliations:** ^1^Laboratory of Metabolic Brain Diseases, Institute of Metabolic Disease Research and Drug Development, China Medical University Shenyang, China; ^2^Henry M. Jackson Foundation Bethesda, MD, United States

**Keywords:** anaerobic glycolysis, brain activation, glutamate-glutamine cycle, glycogenolysis, NADH, neurodegenerative disease, potassium homeostasis, transmitter glutamate

## Abstract

The 1988 observation by Fox et al. (1988) that brief intense brain activation increases glycolysis (pyruvate formation from glucose) much more than oxidative metabolism has been abundantly confirmed. Specifically glycolytic increase was unexpected because the amount of ATP it generates is *much* smaller than that formed by subsequent oxidative metabolism of pyruvate. The present article shows that preferential glycolysis can be explained by metabolic processes associated with activation of the glutamate-glutamine cycle. The flux in this cycle, which is essential for production of transmitter glutamate and GABA, equals 75% of brain glucose utilization and each turn is associated with utilization of ~1 glucose molecule. About one half of the association between cycle flux and glucose metabolism occurs during neuronal conversion of glutamine to glutamate in a process similar to the malate-aspartate shuttle (MAS) except that glutamate is supplied from glutamine, not formed from α-ketoglutarate (αKG) as during operation of conventional MAS. Regular MAS function is triggered by one oxidative process in the cytosol during glycolysis causing NAD^+^ reduction to NADH. Since NADH cannot cross the mitochondrial membrane (MEM) for oxidation NAD^+^ is re-generated by conversion of cytosolic oxaloacetate (OAA) to malate, which enters the mitochondria for oxidation and in a cyclic process regenerates cytosolic OAA. Therefore MAS as well as the “pseudo-MAS” necessary for neuronal glutamate formation can only operate together with cytosolic reduction of NAD^+^ to NADH. The major process causing NAD^+^ reduction is glycolysis which therefore also *must* occur during neuronal conversion of glutamine to glutamate and may energize vesicular glutamate uptake which preferentially uses glycolytically derived energy. Another major contributor to the association between glutamate-glutamine cycle and glucose utilization is the need for astrocytic pyruvate to generate glutamate. Although some oxidative metabolism occurs during glutamate formation it is only one half of that during normal tricarboxylic acid (TCA) cycle function. Glutamate’s receptor stimulation leads to potassium ion (K^+^) release and astrocytic uptake, preferentially fueled by glycolysis and followed by release and neuronal re-accumulation. The activation-induced preferential glycolysis diminishes with continued activation and is followed by an increased ratio between oxidative metabolism and glycolysis, reflecting oxidation of generated glutamate and accumulated lactate.

## Correlation between Brain Glucose Utilization and The Glutamate-Glutamine Cycle

Blood flow to the brain is much higher than corresponding to its volume or weight (Xing et al., 2017). Under resting conditions brain metabolism in the adult brain occurs almost exclusively as complete oxidative metabolism of glucose (Siesjö, 1978; Shulman et al., 2001; Patel et al., 2014) in both neurons and astrocytes, which show similar rates of oxidative metabolism of glucose (reviewed by Hertz, 2011). These two cell types are connected by the glutamate-glutamine cycle, which carries glutamate synthesized in astrocytes to neurons to cover their entire supply of transmitter glutamate and GABA, which cannot be synthesized in neurons (Bringmann et al., 2013; Schousboe et al., 2013; Hertz and Rothman, 2016, 2017). This cycle provides a prime example how specific metabolic processes in different brain cell types are integrated to enable complex behaviors, and it will be described in more detail later. Here it suffices to mention that: (i) its rate is very high since it corresponds to ~75% of the total metabolism of glucose in brain; and (ii) utilization of approximately one glucose molecule occurs during each turn of the cycle (Sibson et al., 1998; Hyder et al., 2013; Hertz and Rothman, 2016).

## Glycolysis Is Preferentially Increased during Brief Brain Activation

Glucose metabolism is a two-stage process in which glucose initially is converted to pyruvate during glycolysis and pyruvate subsequently is completely oxidized in the tricarboxylic acid (TCA) cycle. The amount of pyruvate found in brain is small because most is generally rapidly metabolized, and the small amount present is at the existing redox potential converted to lactate, which is present in at least 10 times higher amount (~1 mM) than pyruvate.

Only four molecules of ATP are formed during glycolysis, and since the initial conversion of glucose to glucose-6-phosphate by hexokinase consumes one ATP molecule and formation of fructose 1,6-bisphosphate from fructose 6-phosphate another ATP the net ATP yield is lowered to two ATP for each glucose molecule. Much more ATP is generated by oxidative metabolism, where the theoretical maximum is 36 ATP, which generally is reduced to 30 due to a less than 100% efficiency of oxidative phosphorylation. Nevertheless, during brief brain activation glucose utilization is preferentially up-regulated compared with oxygen consumption in spite of adequate oxygen supply as discussed below. This can be expressed as a decrease in oxygen-glucose index (OGI), which indicates cerebral metabolic rate for oxygen (CMR_O2_) divided by cerebral metabolic rate for glucose (CMR_glc_), i.e., CMR_O2_/CMR_glc_. Under resting conditions OGI is generally close to (or slightly below) the theoretical value of 6, indicating complete oxidation of glucose almost exclusively via glycolysis to pyruvate and subsequent oxidation of pyruvate in the TCA cycle. This would also *a priori* be expected to be the case during energy-requiring brain activation due to the much higher ATP yield during pyruvate oxidation.

Nevertheless, Fox et al. (1988) showed that intense visual stimulation, which according to information from the author lasted 40s, increases glucose uptake (measured with ^18^F-fluorodeoxyglucose) by 51% and blood flow (measured with ^15^O-labeled water) by 50% in human brain, but oxygen consumption (measured with ^15^O_2_) by only 5%. The increases in blood flow and CMR_glc_ without corresponding increases in CMR_O2_, which this large decrease in OGI indicates, were found highly unlikely by Sokoloff (2008) who claimed that intracellular changes due to increased functional activity which stimulate CMR_glc_ also stimulate CMR_O2_. However, as pointed out by Fox et al. (1988) their finding is consistent with a previously observed increase in lactate in activated brain (Hossmann and Linn, 1987; Prichard et al., 1987), which was later confirmed during visual stimulation (Prichard et al., 1991). Moreover, exit of lactate to extracellular fluid, blood and the lymphatic system has been demonstrated (Dienel and Cruz, 2016) as will be discussed later, and an decrease in OGI in many specific brain regions during brief brain stimulation has been abundantly confirmed (Lear and Ackermann, 1989; Adachi et al., 1995; Madsen et al., 1995, 1998, 1999; Schmalbruch et al., 2002; Cruz et al., 2007), as recently reviewed in detail by Dienel and Cruz (2016). Schmalbruch et al. (2002) made the important additional observation that the decrease in OGI in rat brain is prevented by propranolol, an inhibitor of both β_1_-adrenergic and β_2_-adrenergic receptors. This finding is consistent with previous studies by MacKenzie et al. (1976a,b) that noradrenaline causes a decrease in OGI after intraventricular injection or infusion into the carotid artery of anesthetized baboons with disrupted blood-brain barrier, whereas infusion of the β-adrenergic antagonist propranolol increases OGI. The β_1_-adrenergic receptor stimulates glycogenolysis in brain (Xu et al., 2014; Hertz et al., 2015d), which is important for the glutamate-glutamine cycle because it is required for glutamate synthesis (Gibbs et al., 2007; Obel et al., 2012) and thus synthesis of transmitter glutamate. These transmitters are essential for brain activation, including learning and experience-induced plasticity (Ng et al., 1997; Gibbs and Hertz, 2005; Guzmán-Ramos et al., 2012; Wassum et al., 2012; Hasan et al., 2013; Banks et al., 2014; Barker and Warburton, 2015; Chen et al., 2016; Hertz and Chen, 2016a; Wijtenburg et al., 2017). However, Schmalbruch et al. (2002) did not investigate whether propranolol’s activity was β_1_- or β_2_-mediated.

Part of the mismatch between CMR_glc_ and CMR_O2_ can be explained by the increase in brain lactate during brief stimulation (Sappey-Marinier et al., 1992) and by release of lactate from brain cells (Dienel and Cruz, 2016). During a 6-min brain activation where rats after opening of their cage were stimulated by bilateral gentle stroking with small paint brushes at the face, whiskers, body, forepaws, back, and tail, Madsen et al. (1999) observed a doubling of brain lactate, whereas glycogen decreased, indicating that glycogenolysis had been activated. Activation-induced decrease in OGI is also associated with removal of lactate from the activated region and even from brain cells and the brain itself. Thus, in the auditory pathway unilateral stimulation increases CMR_glc_ in tonotopic bands in the activated inferior colliculus by 35%–85% compared with contralateral tissue, removal of ^14^C-labeled lactate from the inferior colliculus increases by 21% and the levels of extracellular lactate doubles (Cruz et al., 2007). Moreover perivascular routes that drain to lymph nodes (Bradbury and Cserr, 1985) clear large amounts of lactate from the brain during acoustic stimulation (Ball et al., 2010). Lactate release to blood is also increased during brain activity. Thus, the level of arterial lactate increased from 0.5 to almost 2 mM in the experiments by Madsen et al. (1999), and a massive exit of lactate to blood occurs during spreading depression, a situation associated with intense loss of intracellular K^+^ and subsequent Na^+^, K^+^-ATPase-mediated reuptake (Cruz et al., 1999).

**Figure 1:**
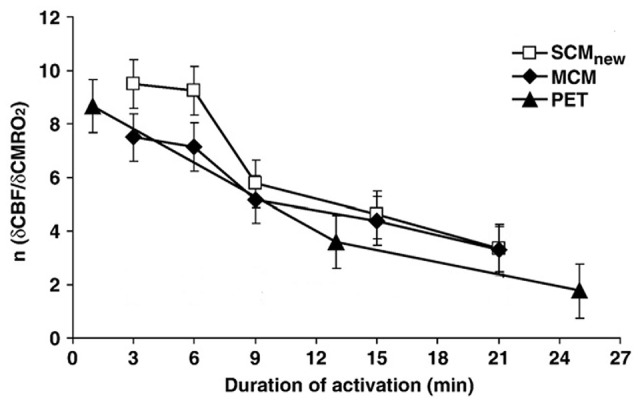
Coupling ratio (*n*) between increase in cerebral blood flow (CBF) in human primary visual cortex (relative cerebral blood flow [δCBF]) and concomitant increase in cerebral rate of O_2_ consumption (relative cerebral metabolic rate of oxygen [δCMR_O2_]), at different times during prolonged intense visual stimulation. Note the consistent decline (*r* = −0.97) in *n* with time, although it does remain positive. The magnetic resonance imaging (MRI) results analyzed by a single-compartment model (SCMnew) and a multiple-compartment model (MCM) were obtained in eight volunteers by MRI in the authors’ laboratory and analyzed using two different procedures for calculations. Virtually identical positron emission topography (PET) results observed by Mintun et al. (2002) are also presented for comparison. Modified from Lin et al. (2009) with permission.

Madsen et al. (1999) also investigated metabolism 15 min after the end of the stimulation and found an increase in OGI to 7.7. A different approach to investigate delayed effects has been to compare alterations in the ratio between relative changes induced by brain activation on rate of CMR_O2_ with those induced on cerebral blood flow (CBF), which was found by Fox et al. (1988) to be altered in parallel with CMR_glc_. Lin et al. (2009) showed that the large initial preferential increase in blood flow and thus in glycolysis is transient and almost abolished after ~20 min (Figure 1). This was done by comparing the relationship between relative cerebral blood flow (δCBF) and relative cerebral metabolic rate of oxygen (δCMR_O2_) during continuous visual stimulation (21 min at 8 Hz) with functional magnetic resonance imaging (fMRI) and simultaneously measuring of blood oxygenation level-dependent (BOLD) signals, CBF and cerebral blood volume (CBV). The δCMR_O2_ was determined by both a newly calibrated single-compartment model (SCM) and a multiple compartment model (MCM), and the results agreed between these two models and with previous positron emission topography (PET) studies by Mintun et al. (2002). However, the time course of relative changes in CMR_O2_ and blood flow coupling in human motor cortex during prolonged intense finger tapping is somewhat different (Vafaee et al., 2012).

During the time interval when visual stimulation preferentially increased CMR_glc_ there was an approximately doubling of the relative rate of CMR_O2_ (Lin et al., 2009), indicating that the preferential increase in CMR_glc_ becomes diminished, but not abolished, during prolonged activation. Measurements of brain glucose and lactate in visual cortex have also indicated a long lasting increase in CMR_glc_ (Prichard et al., 1991; Sappey-Marinier et al., 1992; Chen et al., 1993; Frahm et al., 1996).

## Physiological Processes Contributing to Activation-Induced OGI Decrease

**Figure 2:**
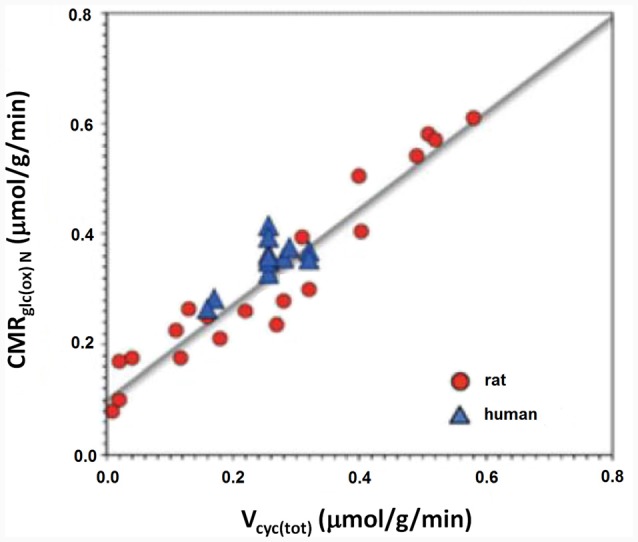
Rate of the glutamate/glutamine cycle vs. glucose oxidation. The measured correlation between the rate of the glutamate/glutamine cycle in rat and human cerebral cortex vs. neuronal glucose oxidation is based on 12 published studies in rodents (red) and nine in humans (blue). The relation is close to linear throughout the entire range with a slope of approximately 1:1. Modified from Hertz and Rothman (2016) with permission.

This part of article will explain why glycolysis *per se* is more important than ATP for cycle flux by attempting to identify metabolic processes contributing to the decrease in OGI during brief brain activation. Several such metabolic processes have been identified as discussed in detail by Dienel and Cruz (2016). Shulman et al. (2001) were the first to consider that the integration between glycolysis and the glutamate-glutamine cycle might play an important role in the reduction of OGI. As shown in Figure 2 approximately one glucose molecule is known to be degraded during the flux of the glutamate-glutamine cycle at all levels of its activity between deep anesthesia and wake activity (Sibson et al., 1998; Hertz and Rothman, 2017).

At the time of Shulman et al.’s (2001) study it was believed that the purpose of this glucose utilization was to provide glycolytically derived energy for glutamate amidation in astrocytes during cycle flux from astrocytes to neurons and for its uptake in astrocytes during the return flux, a concept based on tissue culture experiments by Pellerin and Magistretti (1994) and Pellerin et al. (1998). We now know that this concept is wrong, since glutamate uptake in astrocytes is fueled by oxidation of glutamate itself (McKenna, 2012, 2013; Whitelaw and Robinson, 2013; Jackson et al., 2014). Shulman et al. (2001) hypothesized that additional glucose would have to be utilized if this lactate was formed from glucose via glycogen, i.e., using the glycogen shunt, with release of lactate derived from glycogen, causing OGI to fall. That this might at least partly be the case was suggested by the decreased glycogen content found during early brain activation by Madsen et al. (1999). Metabolism via the glycogen shunt would increase glycolysis because although degradation of glycogen provides one molecule more of ATP per glucose unit than glucose oxidation, a requirement of two ATP per glucose molecule incorporated into glycogen causes glucose flux via glycogen to provide less ATP than glycolysis.

**Figure 3:**
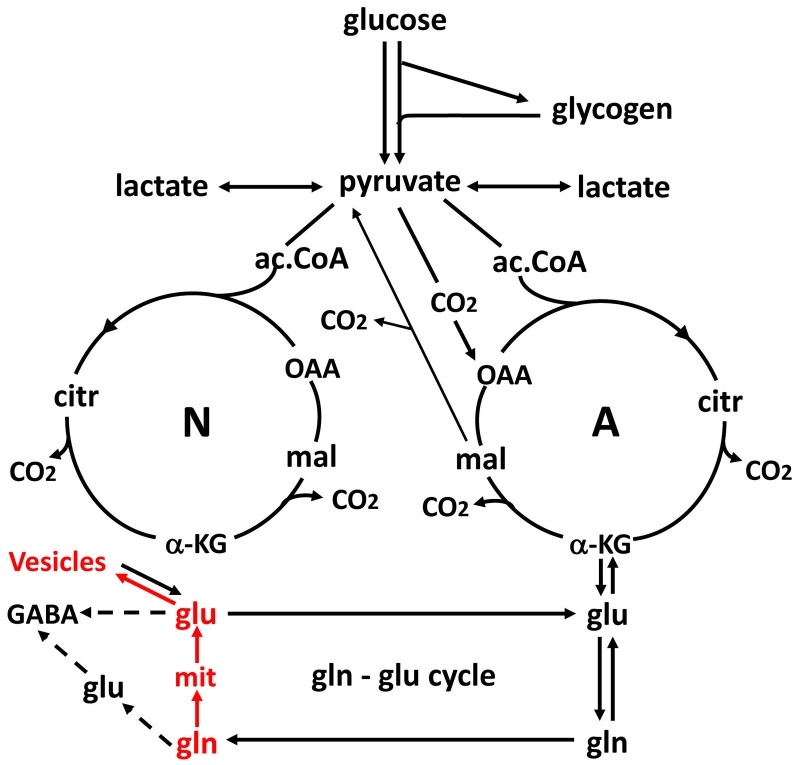
Schematic drawing of glucose metabolism via pyruvate in neurons (left-N) and astrocytes (right-A) and of glutamine-glutamate (GABA) cycling. One molecule glucose is metabolized by glycolysis in the cytosol to two molecules of pyruvate in a complex and strictly regulated pathway, where one oxidative process requires transfer of reducing equivalents to the mitochondria by the malate-aspartate shuttle (MAS). In both neurons and astrocytes pyruvate metabolism via acetyl Coenzyme A (ac.CoA) leads to formation of citrate by condensation with pre-existing oxaloacetate (OAA) in the tricarboxylic acid cycle (TCA), an end result of the previous turn of the cycle. Citrate oxidation in the TCA cycle includes two decarboxylations, leading to production of large amounts of energy (ATP) in the electron transport chain and at the end of the cycle to the re-formation of OAA which enables another turn of the cycle. Pyruvate carboxylation, which is active in astrocytes, but absent in neurons, creates a new molecule of OAA, which after condensation with ac.CoA, derived from a second molecule of pyruvate, forms a new molecule of citrate. This allows utilization of a TCA cycle constituent for glutamate formation and export to neurons in the glutamate-glutamine cycle. The formation of the two molecules of pyruvate requires use of glucose and contributes to the association between the glutamate-glutamine cycle shown in Figure 2 and to activation-induced preferential glycolysis. However, only about 25% of the glutamate/glutamine carried in the cycle is normally newly synthesized, although synthesis can increase during intense stimulation. For glutamate synthesis α-ketoglutarate (αKG), an intermediate of the TCA cycle, is formed from citrate via isocitrate, and the single decarboxylation which is involved reduces but does not abolish the effect on preferential glycolysis, because two decarboxylations occur in the full TCA cycle. αKG can leave the cycle to form glutamate (glu), catalyzed by aspartate aminotransferase. Further metabolism by the cytosolic and astrocyte-specific enzyme glutamine synthetase leads to the formation of glutamine (gln). In glutamatergic neurons all glutamate formed by deamidation of glutamine, shown in red, enters the mitochondria (mit) and is returned to the cytosol in a complex process, which requires simultaneous glucose metabolism, as illustrated in Figure 5. This requirement may explain about one half of the association of the cycle with glucose utilization and must be a major contributor to activation-induced preferential glycolysis. It is followed by accumulation of glutamate into vesicles, also shown in red, which to a major extent uses glycolytically derived ATP. In GABAergic neurons the pathway via mitochondria is only used for some glutamate, from which GABA is formed, whereas the remainder enters the cytosol directly. Released glutamate acts on glutamate receptors to cause neuronal stimulation, accompanied by K^+^ release and subsequent re-uptake initially in astrocytes and subsequently neurons. At least the astrocytic uptake partly depends upon glycolytically derived energy (see Figures 4A,B), which may contribute substantially to activation-induced preferential increase in glycolysis. Extracellular glutamate is almost quantitatively re-accumulated in astrocytes, together with at least part of the released GABA [upper line of the glutamine–glutamate/GABA cycle (glu–gln cycle)] and re-accumulated in the astrocytic cytosol. Here, about 75% is converted to glutamine and re-enters the glutamine–glutamate/GABA cycle. The remaining ~25% is oxidatively degraded, via one of two partly different pathways. In both αKG is reconverted to malate. In one malate exits to the cytosol, is decarboxylated by cytosolic malic enzyme to pyruvate, which is oxidized in the TCA cycle via ac.CoA. Oxidation of glutamate and glutamate-derived pyruvate may explain part of the post-stimulatory increase in oxidative metabolism relative to glycolysis observed by Madsen et al. (1999). In the other malate does not exit the TCA cycle but may be further metabolized to αKG after condensation with ac.CoA, allowing re-synthesis of another molecule of glutamate from only one molecule pyruvate. In either case the degraded glutamate must be replaced by a quantitatively similar production of glutamate from glucose, in the first case by complete *de novo* synthesis from one molecule glucose, in the second from one half of a glucose molecule. Modified from Hertz and Rothman (2016) with permission.

As previously mentioned glycogen metabolism is necessary for the formation of glutamate from glucose (Gibbs et al., 2007; Obel et al., 2012). This is probably in order to support signaling needed for pyruvate carboxylation, which provides the “extra” molecule of TCA constituents used for the astrocytic glutamate synthesis (Figure 3; Hertz and Rothman, 2016), details of which are described in the legend of the figure. The amount of glycogen broken down is probably often small (Khowaja et al., 2015), since it is unlikely that glycogen in the adult mammalian brain is the source of the two pyruvate molecules used for synthesis of one molecule of glutamate. However, the rate of glycogenolysis varies with stimulus and brain region and can be substantial (Dienel et al., 2002). Accordingly the role of the glycogen shunt for the decrease of OGI may be minor. Nevertheless, if glycogenolysis had been added to the glucose consumed and used to re-calculate OGI in the experiments by Fox et al. (1988) and Lin et al. (2009), the CMR_O2_/CMR_glc_ ratio would have fallen even further. More recently a computation by Massucci et al. (2013) has indicated that an increase in glutamate-glutamine cycle flux is associated with a decrease in OGI. This is important, since cycle flux rises in parallel with brain activity (Sibson et al., 1998; Hertz and Rothman, 2016) as was shown in Figure 2. Massucci et al. (2013) also found larger decreases in OGI at higher CMR_glc_. On account of the considerable evidence found for an association between cycle flux and OGI we will below discuss the possible effect of each process in the glutamate-glutamine cycle on: (i) glucose utilization; and (ii) OGI.

**Figure 4:**
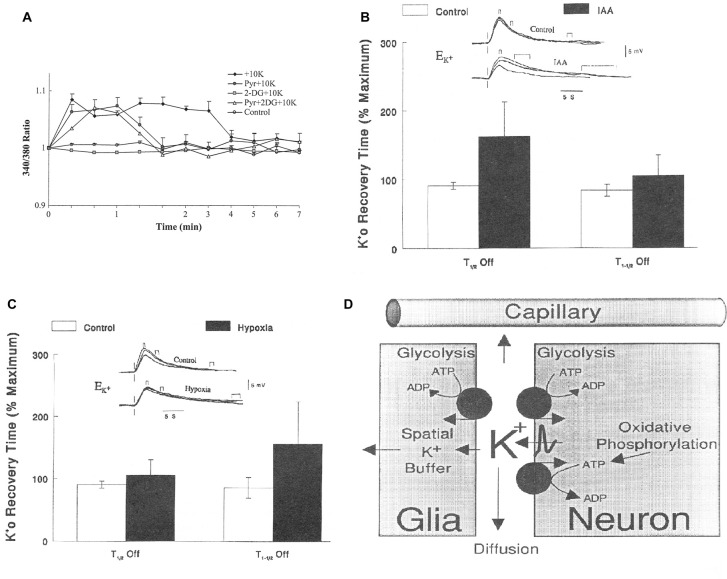
**(A)** Metabolic requirement for K^+^ uptake in cultured astrocytes. Increase in intracellular K^+^ by addition of 10 mM KCl, measured in arbitrary values, by fluorescence of a K^+^-sensitive drug, is abolished by inhibition of glucose metabolism by 2-deoxyglucose (2 DG), but restored by addition of pyruvate. However, the uptake is terminated earlier when glucose metabolism is inhibited and pyruvate is added and even after addition of pyruvate to a medium with glucose (which may decrease glucose utilization), perhaps because K^+^ uptake in the cells requires glycogenolysis (Xu et al., 2014), which depends on glucose metabolism. **(B)** Relationship between half-life for the first (T_1/2_ OFF) and second (T_1–1/2_ OFF) half of the re-uptake of K^+^ and glycolysis in rat brain after increases in extracellular K^+^ concentration (${\text{K}}_{\text{o}}^{+}$), created by varying stimulus intensity and measured using double-barreled, liquid-filled microelectrodes. Increased half-life for re-uptake indicates reduced clearance rate. Top traces: voltage shifts of the K^+^-sensitive microelectrode in response to direct cortical stimulation delivered at the vertical lines at different degrees of K^+^ increase during control conditions and during exposure to 5 mM iodoacetic acid (IAA), a glycolysis inhibitor. Small brackets above the lines indicate maximum increase, medium-size brackets half-maximum return to control values and large brackets full return. Bar graphs: times to K^+^ removal expressed as a percentage of the maximal times in each animal (*n* = 11; the effect of IAA is significant for T_1/2_ OFF (*P* < 0.001) but not for T_1–1/2_ OFF). It could be argued that IAA by inhibiting glycolysis also prevents TCA cycle activity, but IAA slowed K^+^ removal in animals without significant changes in tissue levels of ATP, lactate or pyruvate (Raffin et al., 1992). **(C)** Similar result during severely reduced O_2_ pressure (35–40 mm Hg; *n* = 11; the effect of IAA is now significant for T_1–1/2_ OFF (*P* < 0.001) but not for T_1/2_ OFF). It is now *known* that K^+^ clearance initially occurs into astrocytes and later into neurons and that astrocytic uptake requires glycogenolysis (reviewed by Hertz and Chen, 2016b) rendering it likely that the glycolysis-dependent uptake occurs into astrocytes. That the initial part of the uptake might be astrocytic was also suggested based on the results in **(B,C)** and is shown in **(D)**. This figure is consistent with present knowledge except for the indicated involvement of the Kir.4.1-dependent spatial buffer instead of active transport in the astrocytic K^+^ uptake. **(A)** Modified from Hertz et al. (2015c) with permission; **(B–D)** modified from Rosenthal and Sick (1992) with permission.

The mere formation of the pyruvate molecules needed for synthesis of a “new” TCA constituent requires glycolysis and thus accounts for part of the glucose utilization associated with flux in the glutamate-glutamine cycle. However, because only one quarter of the glutamate carried in the glutamate-glutamine cycle is newly synthesized (Rothman et al., 2011; Hertz and Rothman, 2016), the astrocytic production of pyruvate can under resting conditions at most explain utilization of 0.25 molecule of glucose for each turn of the cycle. This value may be somewhat higher during intense activity which is associated with an increase in glutamate content in the brain (Gibbs et al., 2007; Mangia et al., 2012). Since one of the two oxidations in the TCA cycle (from the 6-carbon compound citrate (or rather isocitrate, which is not shown in Figure 3) occurs before the production of α-ketoglutarate (αKG), the immediate precursor of glutamate, glutamate production explains less of the preferential use of glycolytic energy even during brief brain activation. The one molecule of ATP required for glutamine synthesis contributes little to the association between glucose oxidation and the glutamate-glutamine cycle and is not known to show any preference for glycolytically derived energy. However, the possible uptake of neuronally released NH_4_^+^ by the astrocytic Na^+^, K^+^-ATPase may require some glycolytically derived energy, since K^+^ uptake into cultured astrocytes by the same enzyme is more efficient with glucose as the substrate than with pyruvate as the substrate (Figure 4A), when it lasts for a shorter time, perhaps because glucose is needed for continuous formation of glycogen. Since 2 K^+^ are transported by each ATP the glycolysis associated with NH_4_^+^ must be minute. However, glycolysis is also essential for part of the re-uptake of K^+^ from the extracellular fluid in intact brain (Raffin et al., 1992), as will be discussed later.

**Figure 5:**
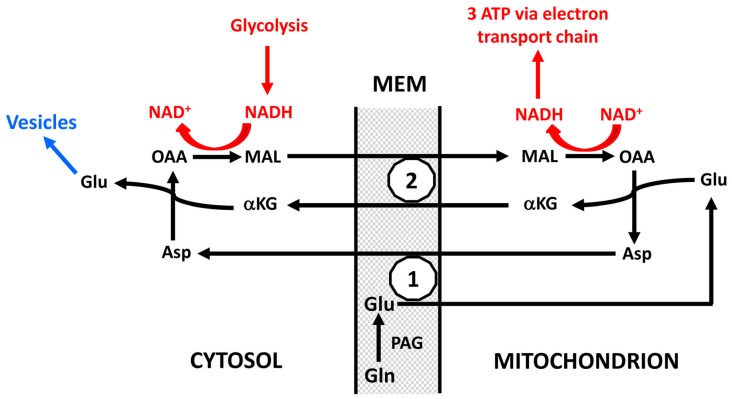
Schematic drawing of cytoplasmic and mitochondrial reactions concluded to occur during biosynthesis of transmitter glutamate from glutamine in the glutamate-glutamine cycle because of inhibition of glutamate synthesis in cultured glutamatergic neurons by the transamination inhibitor aminooxyacetic acid (AOAA) and by phenylssuccinate, an inhibitor of the ketodicarboxylase carrier (Palaiologos et al., 1988, 1989). On the basis of functional considerations phosphate-activated glutaminase (PAG) has been located on the outer surface of the mitochondrial inner membrane as suggested by Kvamme (1983). Generated glutamate (Glu) is carried into the mitochondria across the inner mitochondrial membrane (MEM; the part of the MEM which is impermeable unless specific carriers are utilized) by the aspartate-glutamate carrier (1), in brain aralar, and transaminated to α-ketoglutarate (αKG), which is carried to the cytosol by the ketodicarboxylate carrier (2). At the same time OAA is transaminated in the mitochondria to aspartate which is carried across the inner MEM by the aspartate-glutamate carrier in exchange with the glutamate entering the mitochondria. In the cytosol αKG is re-transaminated to glutamate, with concomitant transamination of aspartate to OAA. After its release in the cytosol this glutamate is accumulated into vesicles to form transmitter glutamate (shown in blue and not part of the process forming glutamate from glutamine), OAA is reduced in the cytosol to malate (MAL) which is carried across the MEM by the ketodicarboxylate carrier in exchange with αKG and in the mitochondria oxidized to OAA. This entire process is identical to the MAS with the exception that glutamate in MAS is not released into the cytosol but is the source of the glutamate which in the Figure is generated from glutamine. The NAD^+^ and NADH shown in red do not participate directly in the formation of glutamate but the oxidation and reduction are necessary cytosolic and mitochondrial processes for the glutamate formation to proceed. Without them OAA could not be converted to malate in the cytosol and malate to OAA in the mitochondria. They are likely to be a result of glycolysis which in many cases also may provide the one molecule of ATP required for the vesicular glutamate uptake shown in blue. The requirement for concomitant glycolysis when glutamine is converted in neurons to glutamate is a major reason for the association of the glutamate-glutamine cycle with glucose utilization and thus probably also for preferential glycolysis during intense brain activation. The process was shown in cultured neurons by Palaiologos et al. (1988, 1989), and supporting evidence was obtained by Bak et al. (2008) in isolated brain mitochondria. Modified from Palaiologos et al. (1988) with permission.

There is evidence that neuronal uptake of glutamate is glycolysis-dependent (Schousboe et al., 2011) but it is not known whether this also applies to glutamine, and even if neuronal glutamine uptake should be glycolysis-dependent the quantitative role must again be minor. In contrast conversion of glutamine to glutamate plays a very major role in preferential utilization of glycolysis. As indicated in Figure 5 and described in more detail in its legend, this is not a simple cytosolic process, but glutamate formed within the mitochondrial membrane (MEM) is initially released into mitochondria (Palaiologos et al., 1988, 1989; Bak et al., 2008). From here it is transferred to the cytosol in a process that is identical to the malate-aspartate shuttle (MAS) with the only exception that glutamine is released within the membrane rather than being formed from αKG in the cytosol. The advantage of this pathway is probably that MAS is a highly regulated process (Del Arco et al., 2016). MAS is better known from glucose metabolism to pyruvate during which there is one oxidative process in the cytosol (conversion of glyceraldehyde-3-phosphate to 1-3-biphosphoglycerate catalyzed by glyceraldehyde phosphate dehydrogenase) which leads to NAD^+^ conversion to NADH. Since these coenzymes cannot cross the MEM, NAD^+^ is re-generated by reduction of oxaloacetate (OAA) in the cytosol to malate which enters the mitochondria in MAS in the same manner as it does in Figure 5 and in a cyclic process is re-converted to OAA. Similar to MAS there must during transfer of glutamate to the neuronal cytosol also be a formation of NADH, normally caused by glycolysis and followed by re-synthesis of NAD^+^ from NADH with simultaneous conversion of OAA to malate. This is probably the major contributor to the large decrease in OGI during the initial part of brain activation, and it accounts for as much one half of the glucose requirement during glutamate-glutamine cycle flux. The evoked glycolysis may support energy requiring neuronal processes, such as vesicular glutamate uptake. This process has also been found to preferentially be driven by glycolysis, and glyceraldehyde phosphate dehydrogenase as well as 3-phosphoglycerate kinase, which forms 3-phosphoglycerate from 1-3-biphosphoglycerate and in the process forms ATP from ADP are enriched in synaptic vesicles (Ikemoto et al., 2003). Energy requirement for vesicular glutamate uptake would thus be ideally suited to supply the NADH molecule needed for conversion of glutamine to glutamate as shown in red in Figure 5. These mechanisms are consistent with the finding by Bak et al. (2008) that formation of [U-^13^C]glutamate from exogenous [U-^13^C]glutamine in brain mitochondria is decreased by inhibition of mitochondrial glutamine uptake with histidine. They are also in agreement with requirement of glucose for synaptic activity in cultured glutamatergic neurons (Bak et al., 2006) and with attenuation of evoked field potentials from dentate granule cells at a low glucose concentration even in the presence of pyruvate (Cox and Bachelard, 1982). Moreover, there is morphological evidence that many presynaptic terminals lack mitochondria (Chavan et al., 2015). Accumulation of glutamate into synaptic vesicles depends on the activity of the vacuolar-type H^+^-ATPase, which drives protons into the lumen by generating a proton electrochemical gradient across the membrane, which drives glutamate into the vesicle (Ikemoto et al., 2003; Farsi et al., 2017). This has the practical consequence that one ATP is required for each molecule of glutamate accumulated into vesicles instead of the 2 K^+^ taken up and 3 Na^+^ extruded for each molecule ATP used by the Na^+^, K^+^-ATPase. Accordingly vesicular glutamate uptake might contribute considerably to the association between CMR_glc_ and the glutamate-glutamine cycle and to preferential CMR_glc_ during brain activation, since it will upregulate glycolysis in neurons, and may generate lactate and contribute to the fall in OGI, depending on its linkage to the MAS and oxidation.

Released glutamate is also likely to contribute to preferential use of glycolytically derived energy since its stimulation of glutamate receptors leads to post-synaptic neuronal stimulation and release of large amounts of K^+^, both in response to the receptor stimulation and during action potential propagation. The subsequent K^+^ re-accumulation occurs initially in astrocytes and subsequently in neurons (Hertz and Chen, 2016b) after exit of astrocytically accumulated K^+^ via Kir4.1 channels (Bay and Butt, 2012). As was shown in Figure 4A the astrocytic uptake is better maintained when glycolytically derived energy is available than when pyruvate is the substrate. This is probably because of the need of glycogenolysis for K^+^ uptake into astrocytes (DiNuzzo et al., 2012, 2017; Xu et al., 2014) and possible glycogen depletion in the absence of glucose. The quantitative importance of glycolysis for K^+^ uptake in the brain is impossible to calculate because glutamate uptake in astrocytes may abolish the need of glycogenolysis for astrocytic K^+^ uptake (Hertz et al., 2015b; Larsen et al., 2016), and large amounts of glutamate are accumulated in astrocytes during brain activation. Nevertheless, there seems also to be a requirement for glycogenolysis (Hertz and Chen, 2016b). Moreover Rosenthal and Sick (1992) have shown that the initial one half of K^+^ re-accumulation after neuronal excitation is greatly delayed by iodoacetate, an inhibitor of glycolysis, but almost unaffected by severe hypoxia (Figures 4B,C), whereas the opposite is true for the second half of the uptake. Based on these results the authors suggested already at this early time that the glycolysis-dependent K^+^ uptake occurred into astrocytes (Figure 4D), a suggestion in complete agreement with Figure 4A and with present knowledge (Hertz and Chen, 2016a).

Glutamate re-accumulation occurs almost exclusively in astrocytes (Danbolt et al., 2016). Its oxidative metabolism via αKG gives rise to the second oxidation in the TCA cycle (Figure 3) where αKG is decarboxylated to 4-carbon compounds, including malate, which can leave the TCA cycle and be converted by malic enzyme to pyruvate which is further metabolized (Hertz and Rothman, 2016, 2017). This process provides a substantial amount of energy (Hertz et al., 2007) without any utilization of glucose. Glutamate metabolism may thus contribute to the increased OGI found by Madsen et al. (1999) after the stimulation and probably also to the reduced increase in OGI found by several authors during long-lasting stimulation. At the same time the content of aspartate is reduced (Mangia et al., 2007, 2012), reflecting that glutamate oxidation via aspartate aminotransferase produces aspartate (Hertz and Rothman, 2017). Increased glutamate content suggests that *de novo* astrocytic glutamate production is increased and thus contributes more to glycolysis than under resting conditions when *de novo* synthesis and degradation of glutamate are similar. Increased *de novo* synthesis of glutamate in astrocytes is also indicated by a higher rate of glucose utilization but not of lactate formation in cultured astrocytes at high glucose concentration (Schousboe et al., 1997). A similar phenomenon has not been reported in intact brain tissue but astrocytic metabolism is less than neuronal metabolism and extracellular glucose concentration is much more easily altered in cultured cells than *in vivo*. On the other hand re-establishment of a reduced glycogen content will increase CMR_glc_ but occurs more slowly than glutamate oxidation (Madsen et al., 1999).

An alternative possibility is that malate does not exit the TCA cycle but is metabolized to αKG after condensation with acetyl Coenzyme A (ac.CoA), allowing re-synthesis of another molecule of glutamate from only one molecule pyruvate. This would reduce but not abolish the relative stimulation of CMR_O2_.

## Possible Relation to Neurodegenerative Disease

Consistent with reduced glucose metabolism during lithium-pilocarpine-induced epileptogenesis (Lee et al., 2012), a recent study from the Zilberter group showed that even a small chronic inhibition of brain glycolysis initiates epileptic seizures (Samokhina et al., 2017). Moreover, reduced glucose metabolism in prodromal and early Alzheimer’s disease (Mosconi et al., 2010; Hertz et al., 2015a) is likely to cause memory impairment via mechanisms described in this article and by Hertz and Chen (2017) long before any cell death occurs. Finally idiopathic Parkinson’s disease and atypical Parkinsonism (multiple system atrophy and progressive supranuclear palsy) are associated with decreases in glucose metabolism with a regional distribution that varies between the three diseases. These observations led Zilberter and Zilberter (2017) to conclude that correcting this metabolic deficiency would be an efficient treatment of neurodegenerative diseases, a proposal that might lead to major progress in the treatment of these devastating diseases.

Presently no therapeutic intervention is known to be able to correct the deficient glucose metabolism in neurodegenerative disease. It has also not been established whether a major reason for the devastating effect of these diseases is the brain’s failing ability to up-regulate glycolysis during its activation. However, they may represent a cruel demonstration by Nature of the importance of the increased glycolysis. Since ketone bodies can substitute for glucose in some but far from all its roles in the glutamate-glutamine cycle (Hertz and Rothman, 2016) it may also explain why diet supplementation with ketogenic compounds (Reger et al., 2004; Henderson, 2008; Hertz et al., 2015a; Cunnane et al., 2016) has a limited therapeutic effect in Alzheimer patients, which might increase with early treatment. This also applies to Parkinson’s disease (Veech et al., 2001; Vanitallie et al., 2005; Hashim and VanItallie, 2014). The amount of ketone bodies used is relatively small, which is consistent with the fact that ketone bodies only to a minor degree can replace glucose in the glutamate-glutamine cycle (Hertz and Rothman, 2016). This contrasts the very large amounts of ketone bodies in the ketogenic diet that can have a therapeutic effect in epilepsy. This apparent paradox can be interpreted as an inhibitory effect on the glutamate-glutamine cycle during the hypoglycemic conditions in patients on ketogenic diet which prevents the ability of the brain to convulse (Hertz et al., 2015a) without affecting the metabolic derangement which initially caused the epilepsy.

## Concluding Remarks

The purpose of the present article has been to provide information about processes occurring during glutamate-glutamine cycle flux that require glycogenolysis without trying to review either this cycle or decrease in OGI during brain activation in detail. However, it should be noted that CMR_glc_ is increased in both neurons and astrocytes. It seems reasonable that the glutamate-glutamine cycle without which glutamatergic activity is impossible, may play a major role during brain activation. The apparent metabolic interaction between neuronal glutamate formation from glutamine and vesicular glutamate uptake is especially intriguing and might on its own account for one half of the glucose utilization during glutamate flux. During cycle flux glycolysis as such is obviously more important than energy production as evidenced by the accumulation and release of lactate. This renders factors such as ADP accumulation which regulate both glycolysis and oxidative metabolism irrelevant for control of increases in blood flow and CMR_glc_. Failing ability to increase glycolysis may be a major factor in neurodegenerative diseases such as Alzheimer’s disease and Parkinson’s disease, a possibility that should be urgently investigated.

## Author Contributions

LH wrote the article and YC edited it.

## Conflict of Interest Statement

The authors declare that the research was conducted in the absence of any commercial or financial relationships that could be construed as a potential conflict of interest.
